# The dysregulation of the immune microenvironment during endometrial intraepithelial neoplasia serves as a marker of endometrial carcinogenesis

**DOI:** 10.3389/fimmu.2026.1749138

**Published:** 2026-02-02

**Authors:** Yingying Peng, Guanglei Zhong, Minqi Zhou, Yuwei Yao, Kejun Dong, Zheng Yang, Lanfen An, Jun Zhang, Jiarui Zhang, Shuo Zhang, Qianqian Tang, Hongbo Wang

**Affiliations:** 1 Department of Obstetrics and Gynecology, Union Hospital, Tongji Medical College, Huazhong University of Science and Technology, Wuhan, China; 2 Cancer Center, Union Hospital, Tongji Medical College, Huazhong University of Science and Technology, Wuhan, China; 3 Department of Plastic Surgery, The First Affiliated Hospital of Anhui Medical University, Hefei, China; 4 Clinical Research Center of Cancer Immunotherapy, Wuhan, China

**Keywords:** carcinogenesis, endometrial cancer (EC), endometrial intraepithelial neoplasia (EIN), molecule subtypes, tumor immune microenvironment

## Abstract

The development of endometrial cancer is a gradual malignant transformation process driven by multiple factors, and the immune microenvironment is closely related to clinical outcomes and immunotherapy responses. Under physiological conditions, the immune microenvironment of the normal endometrium undergoes periodic reshaping under the regulation of estrogen and progesterone, maintaining the balance between immune defense and reproductive capacity. However, continuous exposure to risk factors, such as non-antagonistic estrogen, may trigger endometrial intraepithelial neoplasia. During this period, the immune microenvironment becomes dysregulated, supporting malignant progression. For example, estrogen-stimulated interactions between endothelial cells and macrophages, elevated neutrophil/lymphocyte ratios, and the accumulation of regulatory T cells all combine to cause dysregulation of immune microenvironment. The abnormal immune microenvironment that occurs in the precancerous lesion stage interacts with systemic and genetic carcinogenic factors, ultimately shaping the unique immune microenvironment of each molecular subtype of endometrial cancer. POLE-mutated and MSI-H subtype endometrial cancer are immune-infiltrated tumors, whereas the copy-number high subtype is immune-suppressive tumor and the copy-number low subtype is immune-desert tumor. However, still little is known about the immune dysregulation that occurs during the precancerous stage and its impact on subsequent malignant progression. This review systematically describes the changes in the immune microenvironment during the process from normal endometrium to endometrial cancer, emphasizing that endometrial intraepithelial neoplasia is a key stage of immune imbalance, thus paving the way for early immune intervention and precise immunotherapy.

## 1 Introduction

Uterine corpus cancer is one of the most common gynecological malignancies, accounting for 4.6% of all new female cancer cases worldwide in 2022 (1). Endometrial cancer (EC) constitutes over 83% of reported uterine corpus cancers (2). In the United States, approximately 57% of EC are attributed to overweight and obesity, and endometrioid EC is the predominant histological subtype (3–5). Obesity not only promotes the synthesis of estrogen but also increases its bioavailability, thereby promoting the hyperplasia of endometrial epithelium and increasing the risk of endometrial cancer (3, 5).

In 1983, EC was classified into two histological subtypes by Bokhman (6). Type I EC accounts for approximately 80% to 90%, among which about 80% are endometrioid EC that is related to the excessive proliferation of endometrial cells stimulated by estrogen (7). Type II EC is not dependent on estrogen and primarily manifests as serous or clear-cell histological subtypes (2).

Type I EC begins with continuous endometrial hyperplasia, with the histologically identifiable precancerous lesion defined as endometrial intraepithelial neoplasia (EIN), which is typified by gland/stroma ratio >1, glandular structural disorder, and epithelial cytological changes (8, 9). Clinical studies have shown that EIN diagnosis based on biopsy is associated with a 45-fold increased risk of progressing to EC (10, 11). Therefore, EIN represents a critical step in the developmental process of EC.

Tumorigenesis is a complex biological process that involves a wide range of interactions between tumor cells and the tumor microenvironment (TME). The TME consists of tumor cells, abundant immune cells, cancer-associated fibroblasts, endothelial cells, pericytes, and extracellular matrix components secreted by these cells (12). In normal tissues, the microenvironment inhibits tumor growth, while the TME actively enhances tumor cell proliferation and invasion at the primary site. The tumor immune microenvironment (TIME), as a crucial participant of the TME, can not only exert anti-tumor effects but also produce tumor-promoting effects (13). From immune surveillance to immune evasion, TIME is closely related to tumor progression. During the evolution from normal endometrium to EIN and then to EC, TIME has undergone significant changes, providing potential targets for early intervention (14).

This review systematically summarizes the key changes in the immune microenvironment during the development of EC. We described the molecular factors that drive TIME changes in the continuous process from normal endometrium to EIN to endometrial cancer, investigated their contributions to tumorigenesis, and evaluated their clinical significance.

## 2 Immune microenvironment of normal endometrium

**Figure 1:**
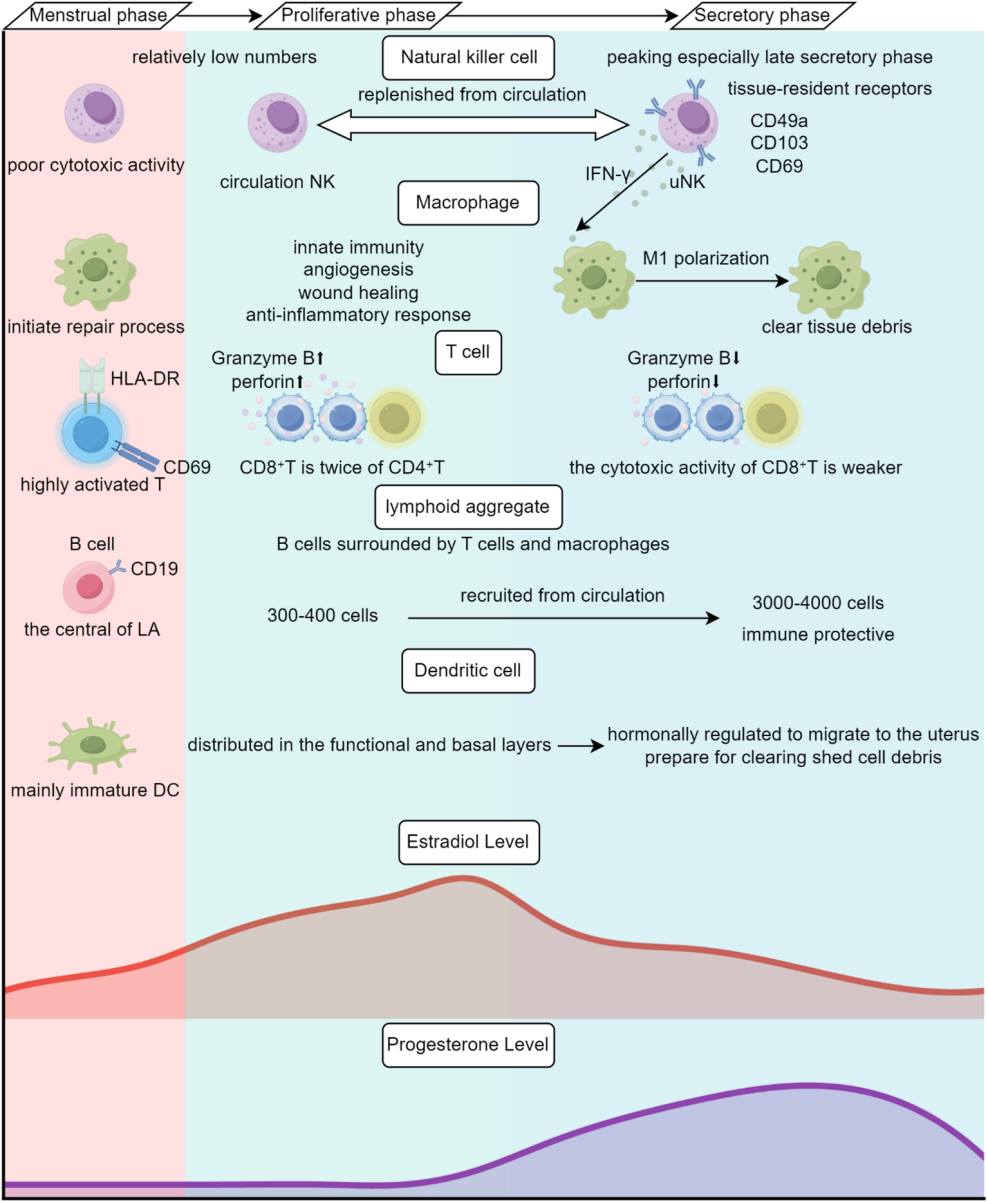
Cyclic remodeling of the immune microenvironment in the normal endometrium driven by estrogen and progesterone. By Figdraw.

Normal endometrium is a unique mucosal immune system (**Figure 1**). It must not only defend against pathogen invasion and clear tissue debris during menstruation, but also tolerate semi-allogeneic embryos and maintain pregnancy (15). Normal endometrium contains various immune cells, some being tissue-resident cells and others migrating from peripheral blood (16).

Uterine natural killer (uNK) cells are the dominant endometrial lymphocytes, with relatively low numbers during the proliferative phase, peaking during the secretory phase (especially late secretory phase), accounting for 70% of lymphocytes in early pregnancy, and decreasing from mid-pregnancy (17–20). uNK cells are equipped with tissue-resident receptors and can be replenished from circulation (21). The classification of uNK cells into endometrial (eNK) and decidual (dNK) subtypes depends on their anatomical location and the host’s pregnancy status (17, 22). Compared with circulating and NK cells residing in other tissues, uNK cells have lower cytotoxic activity (23). They promote uterine spiral artery remodeling through direct interaction with fetal trophoblast cells, secreting proteolytic matrix metalloproteinases (MMP) to provide nutrition for embryonic development (24).

Macrophages are critical to innate immunity and are the main cells for endometrial angiogenesis, wound healing and anti-inflammatory response, which account for 1-2% and 3-5% of the total number of endometrial cells in the proliferative and secretory phases, respectively (25–27). During the secretory phase, macrophages gather around the endometrium and respond rapidly at the onset of menstruation, initiating the repair process (28). Macrophages maintain the homeostasis of the normal endometrium by interacting with other immune cells. For example, during late secretory and early menstrual phases, IFN-γ released by uNK cells promotes M1 polarization of macrophages and enhances their ability to remove tissue debris (29). During pregnancy, macrophages rise to 20-30% of decidual immune cells, exhibiting immunosuppressive properties that favor pregnancy maintenance (30–32).

The decline of T lymphocytes was evident in the endometrium compared with the peripheral blood, accounting for only 1-2% of immune cells, but high expression of HLA-DR and CD69 confirms that these T cells are highly activated (33–35). T cell subsets observed in the endometrium include helper T cells (Th), cytotoxic T cells, regulatory T cells (Tregs), etc., mainly distributed in basal lymphatics, stroma, or epithelium (36). Among them, the proportion of CD8+ T cells is approximately twice that of CD4+ T cells, and the cytotoxic activity of CD8+ T cells is stronger during the proliferative phase and weaker during the secretory phase (37). Additionally, The number of Tregs reaches its peak after embryo implantation to maintain an anti-inflammatory environment and ensures maternal-fetal tolerance (38, 39).

B lymphocytes do not exceed 5% of total lymphocytes in normal endometrium, distributed in endometrial stroma and the central region of lymphoid aggregates (LAs) (40, 41). Current studies on the function of B cells in normal endometrium are limited.

Dendritic cells (DCs) are present in the endometrium at a lower density than NK cells and macrophages, which are mainly immature DCs and are relatively evenly distributed in the functional layer and basal layer of the endometrium (42). During secretory and menstrual phases, some immature DCs are hormonally regulated to migrate to the uterus, preparing for clearing shed cell debris (43, 44). During pregnancy, the presentation of trophoblast-derived fetal antigens by decidual DCs constitutes a fundamental mechanism for maternal immune tolerance (45, 46).

Mast cells (MCs) are abundant in the myometrium but rare in the endometrium, mostly distributed in the basal layer (47). MC phenotypes are related to their location in uterine tissue layers, with tryptase+/chymase+ subset MCs and tryptase-/chymase+ MCs existing in the myometrium and endometrial basal layer, while tryptase+/chymase- MCs exist in the endometrial functional layer (48). The number of MCs in the endometrium shows no significant fluctuation during the menstrual cycle, possibly related to the long lifespan of tissue-resident MCs (49).

Besides immune cells, the endometrial immune system also includes stromal cells and endometrial epithelial cells that mediate antiviral immunity through TLR3 (50). In addition, TLR4 is vital for the innate immune response of endometrial epithelial cells and stromal cells to lipopolysaccharides (51). Moreover, endometrial epithelial cells can express MHC-II, directly process antigens and present them to professional antigen-presenting cells, promoting immune responses (52, 53).

In the endometrial basal layer, there are LAs organized around a core of CD19+ B cells, with T cells and macrophages in the periphery (54). LA size is linked to the menstrual cycle. During the secretory phase, LAs contain 3000–4000 cells, larger than the 300–400 cells during the proliferative phase, more likely derived from immune cells recruited from circulation rather than *in situ* proliferation (55, 56). LAs are similar to tertiary lymphoid structure (TLS), potentially playing an immune protective role during menstruation and disappearing after menopause (41, 55).

With hormonal level changes during the menstrual cycle, the endometrial immune microenvironment is regulated and undergoes cyclical changes (57–59). During the proliferative phase, the level of estrogen gradually increases and reaches its peak before ovulation (60). A variety of immune cells, including B cells, CD4+ T cells, CD8+ T cells, NK cells and DCs, all express estrogen receptors (61). Estrogen receptor α mediates the inhibition of NF-κB pathway, thus high levels of estrogen can promote anti-inflammatory response (62). In addition, estrogen signaling affects the development of innate immune cells, such as driving DCs’ differentiation to enhance local immune surveillance function (63–65). During the secretory phase, the ruptured follicle gradually transforms into the corpus luteum and secretes progesterone to prepare for embryo implantation (66, 67). Progesterone suppresses IFN-γ by binding to progesterone receptors, thereby reducing CD4+ T cell activity (68). Simultaneously, it induces IL-4 production while simultaneously suppressing proliferation in T cells (69). Consequently, the immune reactivity of the endometrium is diminished, further preparing the uterine lining for embryo implantation.

In summary, the immune microenvironment of the normal endometrium undergoes cyclical changes under the influence of estrogen and progesterone, maintaining a delicate state of equilibrium. However, when faced with certain pathological factors, for example, continuous estrogen stimulation, such immune homeostasis is disrupted, thereby creating a breeding ground for precancerous lesions.

## 3 Immune microenvironment remodeling in EIN

**Figure 2:**
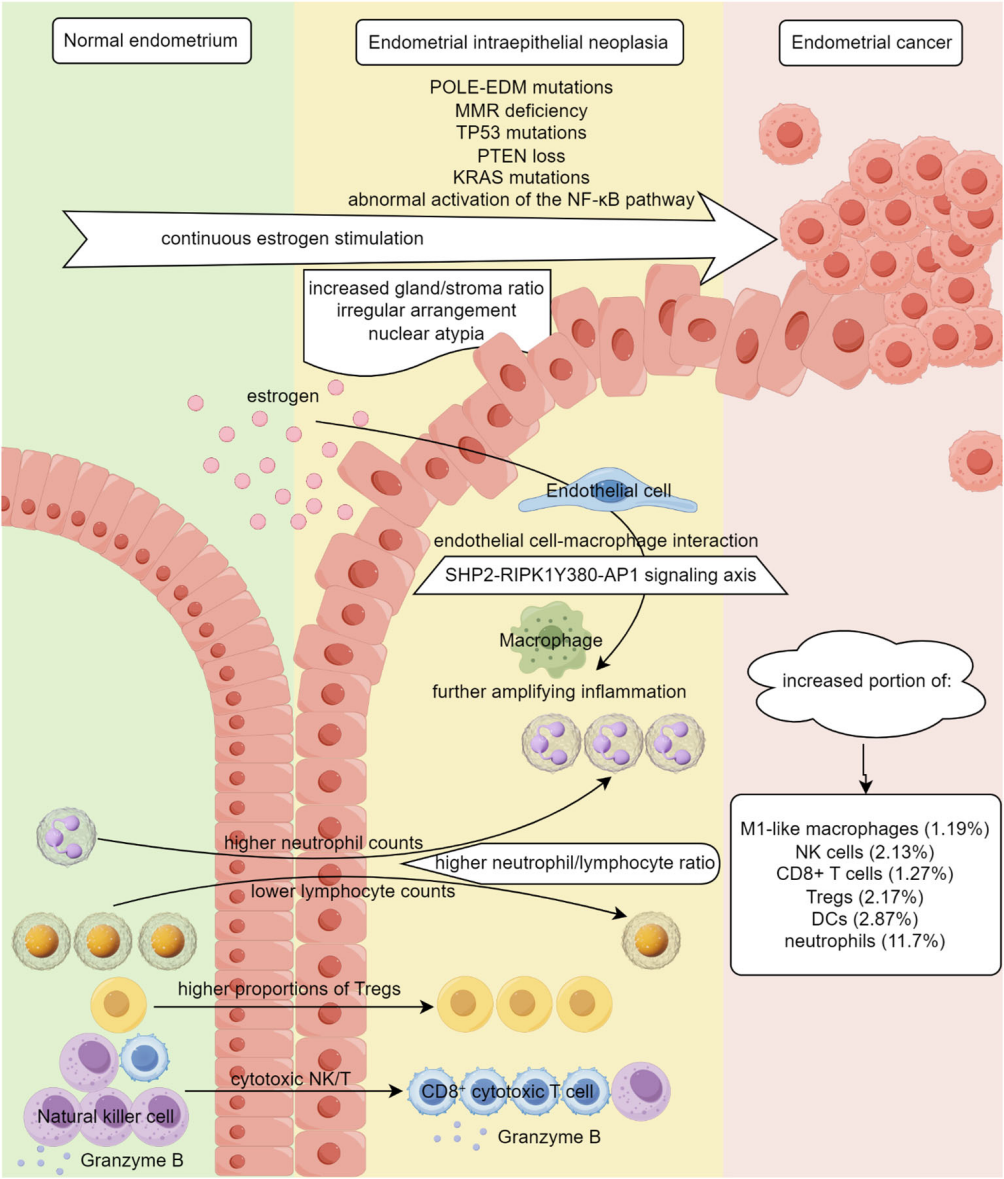
Immune microenvironment dysregulation during the progression of endometrial intraepithelial neoplasia. By Figdraw.

Endometrial intraepithelial neoplasia is the precancerous lesion of type I EC, which is caused by continuous estrogen stimulation, and over 90% of endometrioid ECs are believed to develop from EIN (70). Compared to normal endometrial hyperplasia, EIN is defined as increased gland/stroma ratio, irregular arrangement of cells on the basement membrane, proliferating glands with nuclear atypia (prominent nucleoli and open or vesicular chromatin) (71, 72). EIN carries an extremely high risk of concomitant EC or malignant progression (73, 74). As a critical transition point between precancerous lesions and malignant progression, EIN is an ideal stage for studying early immune changes in Type I EC (**Figure 2**).

During the transition from normal endometrium to EIN, the immune environment undergoes changes, manifested by systemic inflammatory responses and local immune microenvironment alterations.

Peripheral blood inflammatory markers were compared among EC patients, EIN patients, patients with non-atypical endometrial hyperplasia, and normal controls in a retrospective cross-sectional study (75). EIN patients showed higher neutrophil counts than the non-atypical hyperplasia group and control group, lower lymphocyte counts than the control group, and higher neutrophil/lymphocyte ratio (NLR) than all other groups (75). Elevated NLR before treatment predicts poor prognosis in EC patients (76). This study suggests that significant systemic inflammatory responses have already emerged during the EIN phase.

Flow cytometry analysis of endometrial immune cell composition revealed that patients with simple hyperplasia (SEH) had higher total immune cell proportions than proliferative endometrium, while patients with complex hyperplasia (CEH) had lower total immune cell proportions than SEH patients (77). Compared to proliferative endometrium, SEH and CEH patients showed increased effector CD4+ T cell proportions, with SEH patients having elevated neutrophil proportions but reduced NK and NKT cell proportions, and CEH patients showing higher macrophage proportions than SEH patients and proliferative endometrium but lower cytotoxic CD8+ T cell proportions than proliferative endometrium (77). Complex atypical hyperplasia shows an increased Tregs density and a decreased cytotoxic T cells density compared to normal endometrium (78). Moreover, most granzyme B+ cytotoxic NK/T cells within endometrial complex atypical hyperplasia are CD8+ T cells, whereas 80% of cytotoxic NK/T cells in normal endometrium are NK cells (78). In patients with endometrial hyperplasia, continuous estrogen stimulation activates endothelial cells through the SHP2-RIPK1^Y380^-AP1 signaling axis, with macrophages further amplifying inflammation, forming a positive feedback loop of endothelial cell-macrophage interaction communication, thereby maintaining endometrial sterile inflammatory state and causing disease progression (79). In summary, proportional and functional immune alterations are linked to EIN progression.

The changes in the immune microenvironment of EIN may be partly attributed to the driving factors for the transformation of normal endometrium to EIN. PTEN loss and KRAS mutations, which typically emerge at the EIN stage, remodel intercellular crosstalk and reprogram immune-cell recruitment and function through rewired downstream signaling (70, 80, 81). Estrogen exerts potent immunomodulatory effects, and chronic unopposed estrogen exposure reshapes the endometrial immune microenvironment (58, 82). Systemic chronic inflammatory state and insulin resistance caused by obesity may also directly or indirectly affect local immune status in the endometrium (83).

In short, EIN is more than an epithelial premalignancy; it is accompanied by a profound immune dysregulation that may already prime the local microenvironment for progression to cancer.

## 4 Immune microenvironment of EC

The TIME of EC has significant heterogeneity, including immune cells that exert “anti-tumor” functions and perform immune surveillance, as well as immune cells that exert “pro-tumor” functions and perform immune evasion.

### 4.1 Immune cell infiltration in EC

During the development of endometrial hyperplasia into EC, dramatic changes occur in the local immune microenvironment (**Table 1**). Compared to endometrial hyperplasia samples, EC samples show increased proportions of M1-like macrophages (1.19%), NK cells (2.13%), CD8+ T cells (1.27%), Tregs (2.17%), DCs (2.87%), neutrophils (11.7%), while immune checkpoint-related genes (CTLA4, HAVCR2/TIM3, IFNG) expression in EC tends to increase (14, 84, 95, 96).

**Table 1:** Alterations in composition and function of immune cells during progression from normal endometrium to endometrial intraepithelial neoplasia to endometrial cancer.

Cell type	NE	EIN	EC	References
Uterine natural killer cells	relatively low numbers during the proliferative phase peaking during the secretory phase	reduced NK cell proportions	increased proportions of NK cells (2.13%) to EIN while diminished relative to NE increased NK cell infiltration is significantly associated with improved OS	(17–20, 77, 78, 84, 85)
Macrophages	accounting for 1-2% of all endometrial cells during the proliferative phase accounting for 3-5% of all endometrial cells during the secretory phase	amplifying inflammation	TAMs are predominantly M2-like polarized increased proportions of M1-like macrophages (1.19%)	(25–27, 79, 84, 86)
T lymphocytes	accounting for only 1-2% of immune cells	increased effector CD4+ T cell proportions lower cytotoxic CD8+ T cell proportions	increased proportions of CD8+ T cells (1.27%) and Tregs (2.17%) inhibited CD8+ T cytotoxic cell killing effects, reducing GZA, GZB, perforin, and PD-1 expression	(33–35, 37, 77, 84, 87)
B lymphocytes	5% of total lymphocytes	–	increased naive B cell infiltration is associated with longer RFS higher CD20+ B cell infiltration correlates with improved patient prognosis	(40, 41, 88–90)
Dendritic cells	mainly immature DCs	–	increased proportions of DCs (2.87%)	(42, 84, 91)
Neutrophils	–	higher neutrophil counts and higher NLR	increased proportions of neutrophils (11.7%)	(75, 84)
Mast cells	abundant in the myometrium but rare in the endometrium	–	–	(47)
Endometrial epithelial cells	mediate antiviral immunity and express MHC-II	gland/stroma ratio >1 disordered glandular structure epithelial cytological changes	both MHC-I and MHC-II are downregulated in tumor cells approximately 70-80% of EC cells express PD-L1	(8, 9, 50, 92, 93)
Stromal cells	express TLR4 for innate immune response to lipopolysaccharide	–	–	(51)
Lymphoid aggregates	contain 3000–4000 cells during the secretory phase and 300–400 cells during the proliferative phase	–	TLS is found in approximately 19% of high-risk EC patients Higher TLS density is associated with better prognosis	(54–56, 88, 94)

In EC, abundant CD8+ T cells correlate with improved outcomes, and POLE-mut and MSI-H subtypes are also considered more responsive to immunotherapy due to their higher T-lymphocyte infiltration (97–99). However, immunosuppressive cytokines secreted by TME in EC inhibit CD8+ T cytotoxic cell killing effects, reducing their granzyme A (GZA), granzyme B (GZB), and perforin expression (87). CD4+ T lymphocytes include Th and Tregs. Compared to normal endometrium, EC has higher proportions of Tregs, exerting anti-tumor immune suppression (14, 100, 101).

Macrophages exhibit high plasticity in the TME, with polarization being a continuous dynamic process (102). M1-like macrophages usually perform antitumor functions, while M2-like macrophages display anti-inflammatory and tumor-promoting characteristics (103). In EC, tumor-associated macrophages (TAMs) are predominantly M2-like polarized (86).

B lymphocytes also show heterogeneity in TME, with different B cell subtypes exerting pro-tumor or anti-tumor effects, such as regulatory B cells (Bregs) (which may not be a separate lineage) exerting immunosuppressive activity (104). Chemokines drive B cell recruitment into the TME, where B cells further localize to TLS to interact with T cells and antigen-presenting cells (APCs) (105). In EC, higher levels of initial B-cell infiltration correlate with longer relapse-free survival (RFS), and TLS absence independently predicts tumor progression, and higher CD20+ B cell infiltration correlates with improved patient prognosis (88–90). TLS is defined as LAs that can secrete chemokines such as CXCL13, have clear T-cell and B-cell zones, contain high endothelial venules, and the T-cell zone contains mature DCs, while the B-cell zone has germinal centers (106). TLS is found in approximately 19% of high-risk EC patients, with L1CAM as a TLS marker most commonly found in POLEmut and MSI-H subtype patients (94, 107).

The NK cell proportion in EC is lower than that in normal endometrium, while a higher proportion of NK cells is significantly linked to longer overall survival (OS) (85). NK cells comprise two subtypes: CD56^bright^CD16^lo^ and CD56^dim^CD16^hi^, with the former exhibiting immunoregulatory capabilities and cytokine secretion, while the latter primarily kills target cells by secreting perforin and granzyme (108). The cytotoxic effects of CD56^dim^CD16^hi^ NK cells are impaired in EC, which also expresses higher levels of NR4A1 to mediate T cell dysfunction (109, 110). This indicates that NK cell function undergoes significant alterations in endometrial cancer.

Although DCs are more abundant in EC than in normal endometrium, the majority of DCs present in the tumor stroma and margins are immature DCs, which makes tumor antigen presentation ineffective and induces tumor tolerance (91).

In summary, during the progression from normal endometrium to EC, the composition and function of key immune cells undergo dramatic changes, such as impaired cytotoxic function of CD8+ T cells, M2-like polarization of macrophages, NK cell-mediated T cell dysfunction, and ineffective tumor antigen presentation by immature DCs. These changes jointly contributed to the formation of the immunosuppressive microenvironment.

### 4.2 Expression of immune checkpoints

Given the importance of immune checkpoints in modulating immune responses, tumor cells frequently exploit them to evade attack from immune cells (111). Compared with normal endometrial epithelial cell, both classical MHC class I (HLA-A, HLA-B, HLA-C) and class II molecules are downregulated in tumor cells to promote immune evasion, while nonclassical MHC class I molecules (HLA-E, HLA-F, HLA-G) are upregulated to promote immune tolerance (92, 112). In EC, classical MHC class I molecules are downregulated through genetic alterations, epigenetic silencing, or dysregulation of MHC-I transcriptional activator NLRC5 (113, 114). For nonclassical MHC class I molecules, HLA-G is upregulated in EC compared with normal endometrium, which can inhibit the cytotoxicity of NK cells, and low expression of HLA-E predicts improved survival of EC patients (115).

Additionally, approximately 70–80% of EC cells express PD-L1, while 40–70% express PD-L2, resulting in suppressed T-cell activation and function (93). The expression of IDO is more widespread than that of PD-L1 and correlated with reduced infiltration of CD8+ T cells and poor prognosis (116). Additionally, the expression of immune checkpoints on immune cells is also dysregulated. For instance, LAG-3 is more highly expressed in EC with high CD8+ T cell infiltration and it can cooperate with CTLA-4 on Tregs to enhance their immunosuppressive function (117, 118).

### 4.3 Molecular subtypes and TIME characteristics

**Figure 3:**
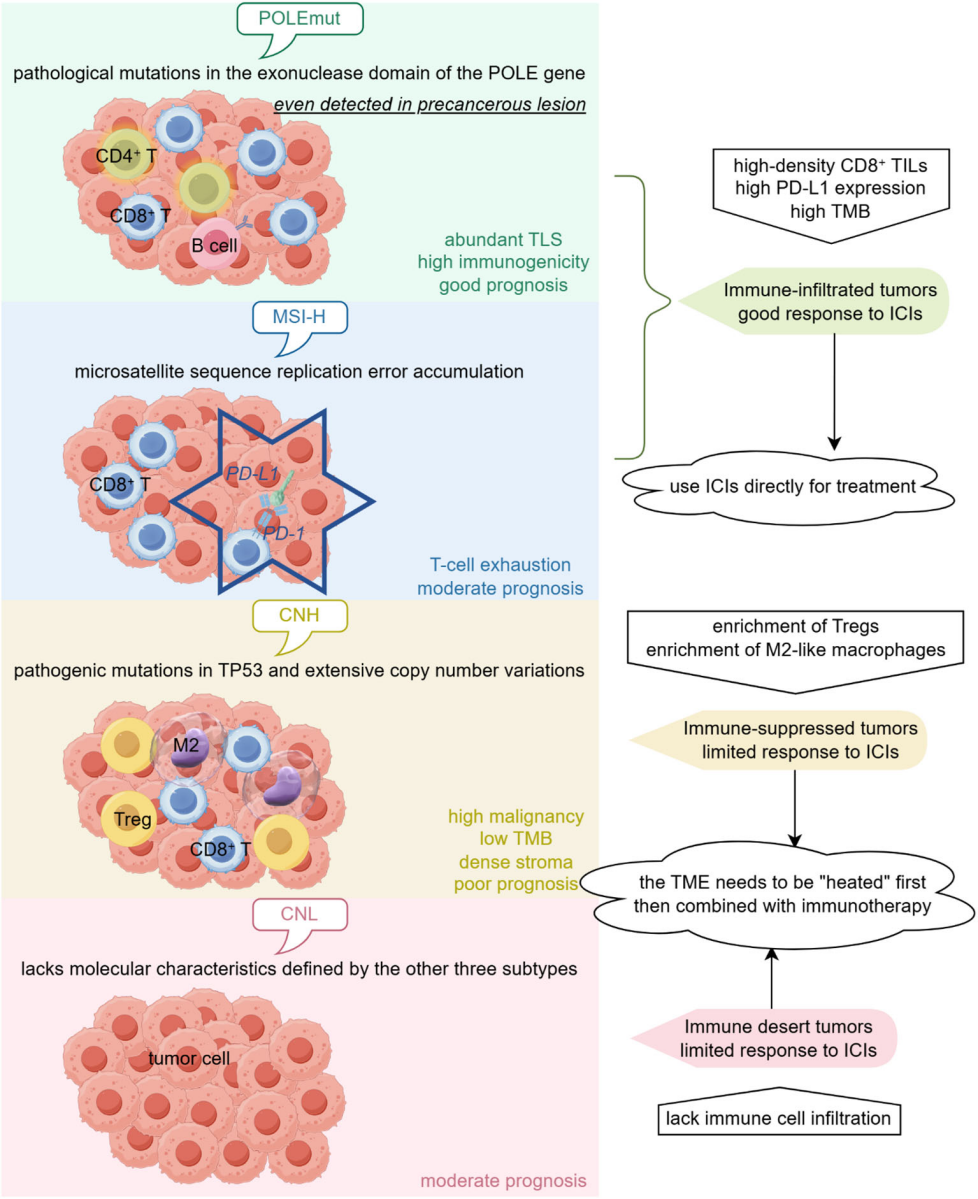
Immunological heterogeneity and therapeutic implications of molecular subtypes in endometrial cancer. By Figdraw.

Based on genomic signatures, The Cancer Genome Atlas (TCGA) categorized EC into four major molecular subtypes, each strongly correlated with obvious TIME features and clinical outcomes (119) (**Figure 3**):

POLEmut EC is recognized by pathological mutations in the exonuclease domain of the POLE gene (encoding the catalytic subunit of DNA polymerase ϵ), which impair mismatch repair function, driving genomic instability and tumorigenesis (120, 121). POLE mutations may even be detected in precancerous lesions (120, 122). The extensive infiltration of T lymphocytes in POLEmut EC enhances its immunogenicity, making such patients susceptible to immune checkpoint inhibitors (ICIs) and generally have a good prognosis (123–125).

Microsatellite Instability-High (MSI-H) EC results from defective DNA mismatch repair, causing microsatellite replication errors, usually accompanied by Microsatellite Instability-High (MMR) protein (MSH2/MSH6/MLH1/PMS2) loss (126). Similar to the POLEmut subtype, MSI-H EC exhibits high tumor mutational burden (TMB), with greater immunogenicity and higher PD-L1 levels compared to other subtypes (127–132). Consequently, it typically responds well to immune checkpoint inhibitor (ICI) therapy. However, a subset of MSI-H EC patients fail to respond to ICI therapy, which may be due to extremely low CD8+ T cell infiltration, impaired terminal T-cell differentiation, a deficiency in mature TLS and DCs, and downregulation of HLA class I in the local immune microenvironment of these patients (133).

Copy-Number High (CNH) EC is defined by pathogenic TP53 mutations, often accompanied by extensive copy number variations (119). CNH EC typically exhibits lower TMB (134). It exhibits high malignancy, frequently presenting as Type II EC or high-grade Type I EC, and is linked to the most unfavorable clinical outcomes (135–139). CD8+ T cells predominantly infiltrate the tumor parenchyma of CNH EC, while their infiltration levels in the tumor stroma are relatively low (132). Conversely, Tregs, M2-like macrophages, PD-L1+CD68+ macrophages, and CD8+PD-1+ T cells exhibit higher proportions, indicating a strongly immunosuppressive microenvironment (132).

Copy-Number Low (CNL) EC lacks molecular characteristics defined by the other three subtypes (140). Its prognosis is intermediate, between TMB-H types (POLEmut and MSI-H) and CNH type (141, 142). CNL EC typically exhibits low intratumoral immune cell infiltration, classified as the “immune desert” type (132, 143, 144).

Among different molecular subtypes of EC patients, TIME composition and functional status exhibit significant differences, including low immune infiltration in CNL EC, relatively active immune microenvironment in CNH EC, while POLEmut and MSI-H EC demonstrate higher CD8+ T cell infiltration levels. Notably, TLSs are organized immune structures composed of diverse immune cells and stromal cells, for instance, lymphocytes, follicular dendritic cells (FDCs), macrophages, and endothelial cells, which play a unique role in the TIME of EC (145, 146). Higher TLS density not only correlates with a better prognosis but also serves as a predictor of ICI treatment responsiveness (88, 147, 148). It is notable that the spatial location of TLS appears to be crucial for its prognostic significance and potential prediction of therapeutic response. Distal TLS (dTLS) significantly prolonged progression-free survival (PFS) across multiple cohorts, and derived greater clinical benefit from ICIs in CNH EC (149).

## 5 Molecular mechanisms underlying immune microenvironment changes

The dynamic evolution of the TIME in EC is jointly driven by the interaction between intrinsic molecular alterations in tumor cells and systemic factors.

Somatic gene mutations are key drivers of immune microenvironment changes. Somatic mutations in the POLE exonuclease correction domain (EDM) were detected in approximately 7%–12% of EC, with 90% of these being pathogenic mutations (119, 150, 151). Mutations at codons 286, 411, 297, 456, and 459 in POLE-EDM are defined as its hotspot mutations, often associated with higher TMB (152, 153). POLE mutations generate more new antigenic epitopes, leading to increased infiltration of CD8+ T cell and enhancement of T cell cytotoxicity and effector molecules such as T-bet, IFN-γ, PRF, and GZB, thereby resulting in higher immunogenicity (154, 155). MMR deficiency accounts for approximately 25%-30% in EC, meaning that it fails to correct insertions or deletions of repeat units during DNA replication through the crucial MMR proteins (156, 157). In MMR-deficient cells, microsatellites are susceptible to replication errors, causing MSI (126, 158). Similar to POLE mutations, MMR deficiency produces high TMB, leading to increased tumor-infiltrating lymphocytes (TILs), although not all patients exhibit this response (133, 159, 160). TP53 is a critical tumor suppressor gene whose encoded protein monitors DNA integrity, initiates cellular responses to DNA damage and triggering apoptosis of abnormal cells (161, 162). Approximately 25%-28% of EC carries TP53 mutations, including one-third of POLEmut EC and 8% of MSI-H EC (119, 163). TP53 mutations are a marker of aggressive disease and poor prognosis (139). TP53-mutated tumor cells induce immune suppression by secreting CSF-1/IL-10/TGF-β to induce M2-like polarization of macrophages, combined with the recruitment of Tregs (162). PTEN gene inactivation is highly prevalent in Type I EC, frequently occurring at the EIN stage (164–167). PTEN loss results in PI3K/AKT/mTOR pathway overactivation and increased cell proliferation (168, 169). PTEN-lost tumors usually show higher densities of Tregs, MDSCs, and M2-like macrophages, inducing an immunosuppressive phenotype (170, 171). KRAS mutations also occur at the EIN stage (172, 173). KRAS gene mutations not only affect the RAF-MEK-ERK pathway, MAPK/ERK pathway, PI3K-AKT-mTOR pathway, and RAL-GEF pathway, thereby promoting cell survival, proliferation, and cytokine secretion, but also facilitate immune evasion by enhancing PD-L1 expression (174, 175).

Besides the direct consequences of gene mutations, abnormal activation or inhibition of key signaling pathways also contribute significantly to immune microenvironment remodeling. The PI3K/AKT/mTOR pathway is frequently activated in EC (commonly due to PTEN loss or PI3KCA mutations), serving as a key driver of tumor cell proliferation, survival, and metabolic reprogramming, and is also associated with chemotherapy resistance (176–178). Besides, this pathway can regulate the composition and function of various immune cells directly or indirectly (179). Abnormal activation of the MAPK/ERK pathway (often caused by KRAS gene mutations) not only affects cell proliferation, differentiation, and stress responses, but is also implicated in chemotherapy resistance (180, 181). Inhibiting this pathway may enhance the antitumor activity of T cells (182–184). As a core regulatory pathway for inflammatory responses, abnormal activation of the NF-κB pathway can not only promote tumor cell survival, metabolism, metastasis, and drug resistance, but also affect Treg development and stability and encoding of pro-inflammatory factors (185). Imbalance in estrogen and progesterone signaling pathways is central to the development of Type I EC. Continuous estrogen stimulation leads to abnormal endometrial hyperplasia, while progesterone resistance impairs fertility-preserving treatment efficacy in young patients (186). Furthermore, estrogen fosters an immunosuppressive microenvironment, which facilitates tumor cell immune evasion, for example, activation of estrogen signaling pathways may promote M2-like polarization of macrophages (187, 188).

The high metabolic demands of rapid proliferation drive metabolic reprogramming in tumor cells, including the Warburg effect (189). In addition to providing energy for tumor cells and supplying substances required for biosynthesis, this metabolic reprogramming also elevates the level of metabolic byproducts like lactate in the TME, which affects the phenotype of infiltrating immune cells (190, 191). Glucose availability in TME is crucial for maintaining NK/T cell function. Under high lactate and low glucose concentrations, effector T cells lose activity, but Tregs, which rely on oxidative phosphorylation, are unaffected (192, 193). Inhibition of glycolysis pathways promotes Treg generation (194). NK cells rely on glycolysis to maintain their activity (195). M1-like macrophages also rely on glycolysis for rapid ATP production (196). In contrast, M2-like macrophages upregulate oxidative phosphorylation and fatty acid oxidation (197). Therefore, tumor cells compete with immune cells in TME, excessively taking up glucose, helping to shape an immunosuppressive microenvironment (198). Similarly, tumor cells also compete with immune cells for amino acids such as glutamine, arginine, and tryptophan, which promotes Treg development, inhibits Th1 differentiation, and impairs NK cell functions (198–202). Metabolic reprogramming of tumor cells also affects lipid content of the TME (202, 203). Tregs can utilize fatty acids in the TME to enhance resistance to PD-L1 therapy (204). Lipid accumulation in DCs impairs their antigen presentation function (205).

Epigenetic mechanisms (such as histone modifications, non-coding RNA regulation, and DNA methylation changes) can cause heritable phenotypic changes without altering DNA sequences, thereby affecting tumor development, progression, and immune microenvironment (206). Methylation of tumor suppressor genes may promote immune defense, while demethylation of oncogenes may promote immune tolerance (206, 207). For example, demethylation of the ERBB2 promoter inhibits effector T cell induction and proliferation (208). Furthermore, non-coding RNAs such as miR-6794-5p can promote M2-like polarization of macrophages via the activation of JAK1/STAT3 pathway, which helps shape an immunosuppressive microenvironment (209).

The TIME is also influenced by systemic factors such as obesity and metabolic syndrome. Weight loss lowers systemic inflammation and promotes the infiltration of protective immune cells into the endometrium (210). Obesity not only elevates circulating estrogen levels but also leads to adipose tissue dysfunction, adipocyte stress, and cell death, ultimately triggering inflammation (211). Compared to EC patients with normal BMI, obese EC patients show increased infiltration of CD3+ T cells and CD163+ macrophages within tumor epithelium, suggesting that the TIME in obese patients may be more disrupted (212).

Molecular factors such as gene mutations, abnormal signaling pathways, and metabolic reprogramming interact with systemic factors like obesity to form a complex regulatory network, jointly shaping the TIME of EC. This network both promotes tumor cell proliferation and suppresses effective antitumor immunity, playing a crucial role in EC progression.

## 6 Role of the immune microenvironment in EC progression

At the earliest stage of tumorigenesis, an influx of naive T cells occurs, potentially performing immune surveillance to recognize and eliminate abnormal cells. However, as the lesion progresses, it rapidly transitions to an immunosuppressive microenvironment. Immune cell subsets undergo transformation, and genes involved in immune suppression are upregulated (12). The persistent chronic inflammatory state, immune-suppressive factors produced by tumor cells, and interactions among other components within the TME collectively shape an immunosuppressive TIME that promotes tumor growth, invasion, and metastasis.

Immune cell subsets such as Tregs and TAMs are known to play a critical role in shaping the tumor-promoting TME. Tregs mediate immune suppression through multiple mechanisms, including: blocking APCs (such as DCs) from delivering stimulatory signals to conventional T cells by overexpressing CTLA-4, LAG-3, and TIGIT; suppressing T cell development and function through the release of IL-10, IL-35, and TGF-β; directly killing CD8+ T cells by secreting granzymes and perforin (213, 214). TAMs usually polarize toward M2-like phenotype in TME, not only promoting tumor vascularization by secreting VEGF, but also promoting tumor invasion and metastasis by secreting factors such as MMP19 (85, 215). Furthermore, M2-like TAMs can recruit Tregs, exacerbate NK cell exhaustion, and suppress CD8+ effector T cell activity (216, 217). CD19+ B cells and CD138+ plasma cells exert anti-tumor effects by secreting IgA, which binds to plgR on tumor cells and triggers apoptosis (218).

CAFs, endothelial cells, and other non-immune cells also participate in tumor progression and immune regulation. CAFs show heterogeneity, including inflammatory CAFs (iCAFs), matrix CAFs (mCAFs), vascular CAFs (vCAFs), antigen-presenting CAFs (apCAFs) subtypes, respectively playing roles in creating immunosuppressive microenvironment, remodeling extracellular matrix, promoting angiogenesis, and participating in antigen presentation (219). Endothelial cells in EC are also heterogeneous, including lymphatic endothelial cells and vascular endothelial cells. These cells may acquire a malignant phenotype through the MDL-NCL signaling pathway derived from tumor epithelial cells (220).

In summary, the interaction between tumor cells, immune cells, and other stromal cells disrupts immune homeostasis through alterations in various molecular signals. Immune escape gradually overpowers immune surveillance functions, collectively shaping a microenvironment conducive to tumor growth. This ultimately enables tumor cells to overcome proliferation constraints, acquire invasive and metastatic capabilities, and promote the progression of EC.

## 7 Clinical significance of TIME in EC

The dynamic evolution of the immune microenvironment during the progression of EC holds significant clinical importance, particularly in assessing patient prognosis, predicting the efficacy of immunotherapy, and guiding immunotherapy strategies.

The characteristics of TIME are closely related to clinical outcomes in EC patients. As mentioned earlier, high-density CD8+ TILs and TLS are usually associated with better prognosis, while enrichment of Tregs and TAMs subsets may lead to worse prognosis. Among the four molecular subtypes of EC, POLEmut type has the best prognosis, followed by MSI-H and CNL types, with CNH type having the worst prognosis, which is largely related to their immune microenvironment characteristics (see Section 4: Immune Microenvironment of EC for details).

The characteristics of TIME serve as an important biological foundation for predicting immunotherapy efficacy. Immune-infiltrated tumors are characterized by high-density CD8+ TILs, high PD-L1 expression, and high TMB, responding well to ICIs, including MSI-H and POLEmut EC (203). Immune-suppressed tumors are characterized by enrichment of Tregs and M2-like macrophages, such as CNH EC (132). Immune desert tumors lack immune cell infiltration, such as CNL EC (143). Therefore, CNH and CNL EC have limited responses to ICI therapy.

The characteristics of TIME can guide immunotherapy strategies. For immune-infiltrated tumors, ICIs can be used directly for treatment; for immune-suppressed and immune desert tumors, the TME needs to be “heated” first, then combined with immunotherapy (203). Specifically, strategies can be employed to target immunosuppressive cells such as TAMs, including their elimination, inhibition of recruitment, reprogramming to M1-like phenotype, and restoration of their phagocytic capacity (221). Extracellular matrix (ECM) can also be remodeled to enhance drug delivery and immune cell infiltration by degrading the ECM using hyaluronidase, targeting pro-tumor subtypes of CAFs, and reducing ECM stiffness (222). Apart from these, therapies such as anti-angiogenesis, metabolic interventions, tumor vaccines, and oncolytic viruses can also improve the immune microenvironment and enhance the efficacy of immunotherapy (203).

## 8 Conclusion

This review systematically summarizes the dynamic changes of the immune microenvironment during the progression from normal endometrium through EIN to EC. The immune system of the normal endometrium is precisely regulated by estrogen and progesterone, undergoing periodic changes throughout the menstrual cycle to adapt to reproductive demands. During the EIN phase, the immune microenvironment begins to remodel, manifested by elevated systemic inflammatory markers and altered proportions of local immune cell subsets, suggesting an immune response to precancerous lesions and potential immune dysfunction. The TIME of EC shows high heterogeneity, and different subtypes exhibit different immune infiltration patterns. Immune cells (including T cells, macrophages, NK cells, DCs, B cells, TLS, etc.) and other stromal components (including fibroblasts, endothelial cells, ECM, etc.) play dual roles in promoting and suppressing tumors during this process, often related to molecular signals in the microenvironment. Molecular mechanisms driving immune microenvironment changes involve tumor cell gene mutations, abnormal signaling pathways, metabolic reprogramming, and systemic factors (such as obesity).

However, the causal relationship between the immune microenvironment and epithelial malignant transformation remains to be fully elucidated. On the one hand, an abnormal immune microenvironment can serve as a driving factor for carcinogenesis. Firstly, chronic inflammation activates pathways such as NF-κB, leading to DNA damage and abnormal proliferation, increased macrophage infiltration, and dysfunction of Tregs (223–225). Secondly, endometrial dysbiosis can also disrupt the immune surveillance function, which facilitates the malignant transformation of endometrial epithelial cells (226, 227). Thirdly, multiple studies have confirmed that the immunosuppressive phenotype emerges earlier than the pathological carcinogenic features, suggesting that it exerts a promoting effect on cancer at the precancerous lesion stage (228, 229). On the other hand, tumor cells can exacerbate carcinogenesis by actively shaping the immune microenvironment. Tumor cells can not only secrete immunosuppressive factors such as TGF-β and IL-10 to recruit inhibitory immune cells such as M2-like macrophages and Tregs, but also down-regulate the expression of MHC class I molecules to evade immune surveillance (230). Consequently, tumor cells and the immunosuppressive microenvironment form a bidirectional positive feedback loop.

Despite significant progress in EC immune microenvironment research in recent years, there are still some limitations. First, detailed characterization of the immune microenvironment at the EIN stage, especially the functional status of different immune cell subsets, remains limited. More research is needed to compare immune characteristics between normal endometrium, EIN (stratified by whether EC is present), and EC. Second, the spatial structure within the tumor microenvironment, interactions between immune cells, and positional relationships need further exploration. Third, within the same immune cell type, there may be multiple functionally different subsets (such as M2-like macrophages including M2a, M2b, M2c, M2d subsets), and the dynamic changes and specific functions of these subsets during EC progression need more detailed elucidation (231). Fourth, EC immune microenvironment needs to be combined with ICI resistance mechanisms for research to more precisely identify responsive populations and develop more effective treatment strategies. Fifth, although patient-derived EC organoid models have been developed, they lack complete immune systems and still cannot fully simulate the immune microenvironment, which limits further research on the immune microenvironment.

Therefore, future research should focus on the following directions. First, widely apply single-cell multi-omics technologies and spatial transcriptomics to map the immune microenvironment of normal endometrium to EIN to various molecular subtypes of EC with higher resolution, revealing spatial distribution of immune cells, different functions, and interaction networks. Second, construct preclinical models that better reflect the complexity of EC immune microenvironment for mechanism research and development of novel treatment strategies. Third, deeply study driving factors and key changes in the immune microenvironment at the EIN stage, exploring the possibility of immune intervention at the precancerous stage to prevent carcinogenesis. Fourth, search for immune characteristics valuable for EC prognosis based on multi-omics data and clinical cohorts. Fifth, explore more personalized combination treatment strategies based on EC molecular subtypes and individual immune microenvironment characteristics.

In conclusion, future advances will depend on in-depth understanding from multiple dimensions of the complex interactions between various cells and specific mechanisms of immune system dysfunction during EC development and progression, ultimately achieving precise and effective immunotherapy strategies based on individual patient characteristics.

## Glossary

EC: Endometrial Cancer
EIN: Endometrial Intraepithelial Neoplasia
AEH: Atypical Endometrial Hyperplasia
TME: Tumor Microenvironment
TIME: Tumor Immune Microenvironment
uNK: Uterine Natural Killer
eNK: Endometrial NK
dNK: Decidual NK
MMP: Matrix Metalloproteinases
Tregs: Regulatory T Cells
LAs: Lymphoid Aggregates
DCs: Dendritic Cells
MCs: Mast Cells
+: Positive
-: Negative
TLS: Tertiary Lymphoid Structure
NLR: Neutrophil/Lymphocyte Ratio
SEH: Simple Hyperplasia
CEH: Complex Hyperplasia
GZA: Granzyme A
GZB: Granzyme B
Th: Helper T Cells
TAMs: Tumor-Associated Macrophages
Bregs: Regulatory B cells
APCs: Antigen-Presenting Cells
RFS: Relapse-Free Survival
OS: Overall Survival
CTLA-4: Cytotoxic T Lymphocyte-Associated Antigen 4
TCGA: The Cancer Genome Atlas
ICIs: Immune Checkpoint Inhibitors
TMB: Tumor Mutational Burden
CNH: Copy-Number High
CNL: Copy-Number Low
FDCs: Follicular Dendritic Cells
FRCs: Fibroblastic Reticular Cells
dTLS: Distal TLS
PFS: Progression-Free Survival
EDM: Exonuclease Correction Domain
TILs: Tumor-Infiltrating Lymphocytes
CAFs: Cancer-Associated Fibroblasts
iCAFs: Inflammatory CAFs
mCAFs: Matrix CAFs
vCAFs: Vascular CAFs
apCAFs: Antigen-Presenting CAFs
ECM: Extracellular Matrix.
MSI-H: Microsatellite Instability-High
MMR: Mismatch Repair
